# Osteoporosis and osteoarthritis: a bi-directional Mendelian randomization study

**DOI:** 10.1186/s13075-023-03213-5

**Published:** 2023-12-13

**Authors:** Yudun Qu, Shibo Chen, Mengling Han, Ziqi Gu, Yujie Zhang, Tianxiang Fan, Muhui Zeng, Guangfeng Ruan, Peihua Cao, Qian Yang, Changhai Ding, Yan Zhang, Zhaohua Zhu

**Affiliations:** 1 Clinical Research Centre, Zhujiang Hospital, The Second School of Clinical Medicine, Southern Medical University, Guangzhou, Guangdong China; 2 Department of Orthopedics, Zhujiang Hospital, Southern Medical University, Guangzhou, Guangdong China; 3 Department of Rehabilitation, Zhujiang Hospital, Southern Medical University, Guangzhou, China; 4 Department of Epidemiology, School of Public Health, Southern Medical University, Guangzhou, Guangdong China; 5 Department of Rheumatology, Guangzhou First People’s Hospital, School of Medicine, South China University of Technology, Guangzhou, 510180 China; 6 MRC Integrative Epidemiology Unit at the University of Bristol, Bristol, UK; 7 Population Health Sciences, Bristol Medical School, University of Bristol, Bristol, UK; 8 Department of Orthopaedics, Affiliated Hospital of Youjiang Medical University for Nationalities, Baise, 533000 China

**Keywords:** Osteoporosis, Osteoarthritis, Mendelian randomization, Gene ontology enrichment analyses, Expression quantitative trait locus analyses

## Abstract

**Objective:**

To investigate the causal relationship between low bone mineral density (BMD) and osteoarthritis (OA) using Mendelian randomization (MR) design.

**Methods:**

Two-sample bi-directional MR analyses were performed using summary-level information on OA traits from UK Biobank and arcOGEN. Sensitivity analyses including MR-Egger, simple median, weighted median, MR pleiotropy residual sum, and outlier approaches were utilized in conjunction with inverse variance weighting (IVW). Gene ontology (GO) enrichment analyses and expression quantitative trait locus (eQTL) colocalization analyses were used to investigate the potential mechanism and shared genes between osteoporosis (OP) and OA.

**Results:**

The IVW method revealed that genetically predicted low femoral neck BMD was significantly linked with hip (*β* = 0.105, 95% CI: 0.023–0.188) and knee OA (*β* = 0.117, 95% CI: 0.049–0.184), but not with other site-specific OA. Genetically predicted low lumber spine BMD was significantly associated with OA at any sites (*β* = 0.048, 95% CI: 0.011–0.085), knee OA (*β* = 0.101, 95% CI: 0.045–0.156), and hip OA (*β* = 0.150, 95% CI: 0.077–0.224). Only hip OA was significantly linked with genetically predicted reduced total bone BMD (*β* = 0.092, 95% CI: 0.010–0.174). In the reverse MR analyses, no evidence for a causal effect of OA on BMD was found. GO enrichment analysis and eQTL analysis illustrated that *DDN* and *SMAD-3* were the most prominent co-located genes.

**Conclusions:**

These findings suggested that OP may be causally linked to an increased risk of OA, indicating that measures to raise BMD may be effective in preventing OA. More research is required to determine the underlying processes via which OP causes OA.

**Supplementary Information:**

The online version contains supplementary material available at 10.1186/s13075-023-03213-5.

## Introduction

Osteoporosis (OP) is a common metabolic skeletal disease characterized by reduced bone mineral density (BMD), resulting in an increment of bone fragility among the elderly [1]. It causes considerable emotional, physical, and financial stresses to patients, and often leads to disability and poor quality of life. Osteoarthritis (OA) is the most common joint disease in which degenerative changes in joint cartilage cause aseptic inflammation and involve the entire joint tissues [2]. Its symptoms include joint pain, stiffness, and activity limitation. The etiology and pathological changes in OA remain largely unclear, resulting in the notable absence of curable therapies.

Does OA directly affect OP, or vice versa? Numerous observational studies have shown a strong link between OP and OA. Some have shown that patients with OA had a lower BMD, but others have reported that the progression of OA was accompanied by an increase in BMD [3]. There is evidence of greater systemic BMD in OA patients at several joint locations, after adjustment for spinal osteophytes [4]. A longitudinal study, however, has concluded that the development of pre-existing OA may be negatively correlated with BMD. Increased age-specific femoral neck BMD quartiles were linked to a lower risk of knee OA development throughout the course of the 8-year trial, according to research by Zhang et al. in the Framingham population [5]. Additionally, Hart et al. noted a tendency for lower hip BMD in those with progressing knee OA compared to non-progressors in the Chingford Study [6]. Although many studies have attempted to explore this controversy, previous observational data are limited for causal inference due to potential biases introduced by confounders. Mendelian randomization (MR), an emerging approach to evaluate causal links, has been popular in recent years. MR may reduce confounding effects and eliminate the bias of reverse causation since genotypes are independent of postnatal lifestyle and environmental variables and predate the illness process [7].

Pleiotropy may exist between OA-influencing variables such as body mass index (BMI) and genetic variables linked to BMD. Using a Mendelian randomization approach, April Hartley et al. found causal effects of hip and knee OA on BMD independent of BMI, while Liu Lin et al. found that OP reduced the incidence of OA (knee OA and hip OA). However, it is still unknown if there are site-specific causal links between low BMD and OA, and the potential signaling pathways involved are not clearly defined. Therefore, the aims of our study were to assess the causal relationship between OP and OA in the framework of a two-sample bi-directional MR study in which multiple OP measures (FN-BMD, LS-BMD, and TB-BMD) and OA measures (OA at any sites, knee OA and hip OA) will be used, and to further explore the potential signaling pathways and shared genes between OP and OA were identified.

## Methods

### Study design and data sources

**Fig. 1:**
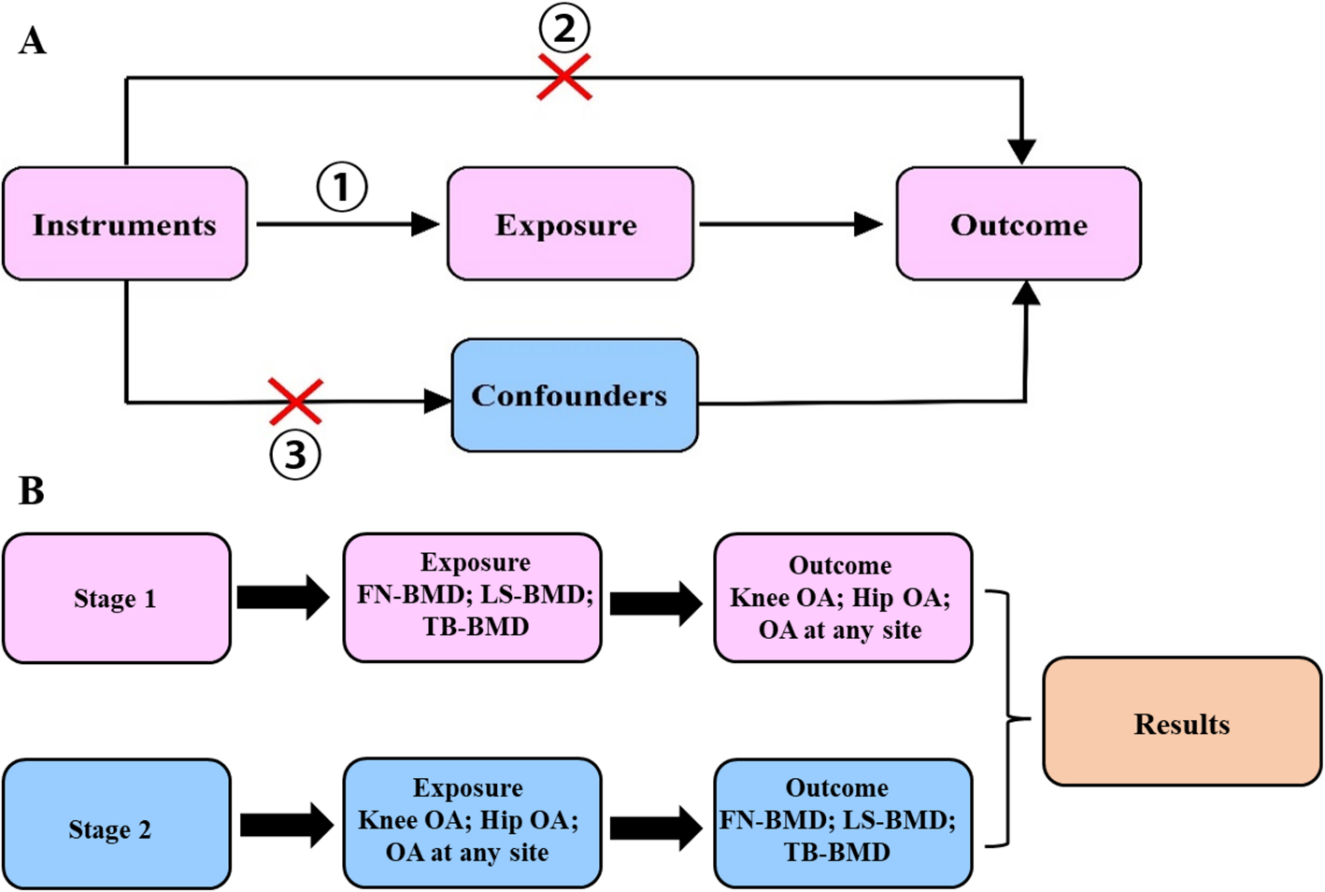
Workflow of bi-directional MR analysis. **A**, the fundamental idea of MR analysis: If we cannot randomize the exposure, we can find a randomized instrumental variable to disentangle. ① Instrumental variables were highly correlated with exposure factors. ② Instrumental variables were independent of the outcome. ③ Instrumental variables were not correlated with confounding factors. **B**, Workflow of our bi-directional MR analysis. MR: Mendelian randomization; BMD: bone mineral density; OA: osteoarthritis; FN: femoral neck; LS: lumbar spine; TB: total body

The causal link between low BMD at each location and OA with various symptoms was investigated in a two-sample bi-directional MR investigation (Fig. 1). GWAS summary statistics of low BMD traits were extracted from publicly available data [GWAS catalog https://www.ebi.ac.uk/gwas/publications/26367794 for femoral neck (FN) [8] and lumber spine (LS) [8], and GWAS catalog https://www.ebi.ac.uk/gwas/publications/29304378 for total body (TB) [9]]. The summary statistics of OA were obtained from the most recent version from GWAS of UK Biobank data [10] (GWAS catalog https://www.ebi.ac.uk/gwas/publications/30664745 and https://msk.hugeamp.org/downloads.html) [11]. The latter summary statistics were used for sensitivity analysis. All data sources were publicly available, and thus, no ethical approval was required.

### Instrumental variables for BMD and OA

OP is defined clinically through the measurement of BMD (*T* score <  − 2.5), which remains the single best predictor of fracture [12]. In this research, BMD was used to characterize three phenotypes of OP measurements at various locations, including FN (*n* = 32,735), LS (*n* = 28,498), and systemic TB (*n* = 66,628). For the three BMD phenotypes, genetic differences in GWAS for poor BMD (− 2.5 T score as cutoff) were employed as instrumental factors. Based on two recent GWAS, we selected instrumental variables for FN-BMD, LS-BMD, and TB-BMD in our main analysis as independent genetic variants at the study-specific genome-wide significance level (*P* = 5 × 10^−8^) [8, 9]. The Genetic Factors for Osteoporosis (GEFOS) that published a meta-analysis of 32,735 FN-BMD and 28,498 LS-BMD in the European population in 2012 were included [8]. The other was the largest meta-analysis to date on FN- and LS-BMD, including 17 GWAS and 32,961 individuals of European descent [13]. Three BMD (per SD) phenotypes were adjusted for age, sex, weight, and height in the previous GWAS studies. Summary statistics for a GWAS meta-analysis research comprising 66,628 European participants were included in the GWAS dataset for TB-BMD [9]. From the genomic-wide meta-analysis spanning the UK Biobank and arcOGEN with 384,838 Europeans, summary-level data for three OA phenotypes, including OA at any sites, knee OA, and hip OA, were collected [10]. Sensitivity analysis was performed using the latest OA databases released in 2021, which conducted a GWAS meta-analysis containing 826,690 individuals (177,517 with OA). They discovered 100 risk mutations across 11 OA phenotypes that were independently linked with the illness, 52 of which were previously unrelated to the condition [11].

Genetic variations linked to low BMD were considered as instrumental SNPs in the forward stage (FN-BMD, LS-MD [8], and TB-BMD [9]). We used linkage disequilibrium (LD) [14] clustering based on r^2^ > 0.001 and removed variations within 1 Mb distance from other SNPs with a greater connection to fulfill the independence across instrumental SNPs for each exposure. Additionally, we harmonized the impact of these instrumental SNPs where possible and eliminated those that were absent from the GWAS of the outcomes to make sure that all associated risk factors and result alleles were on the same strand. Then, SNPs associated with low BMD were selected (FN-BMD 47, LS-BMD 47, TB-BMD 43) (Table S1). In the reverse stage, genetic variants associated with OA were used as instrumental SNPs [10]. Similarly, we used LD clumping based on *r*^2^ > 0.001 and removed variations within 1 Mb distance from other SNPs with a strong correlation to guarantee independence across instrumental SNPs for each exposure. To ensure that all corresponding risk factors and outcome alleles were on the same strand, we harmonized the effect of these instrumental SNPs where possible, and those not present in the GWAS of the outcomes were removed. At the same time, SNPs that might have horizontal pleiotropy were removed and the frequency of efficient alleles was used to ensure that palindrome instruments were correctly aligned where possible. Finally, SNPs associated with OA were selected (OA at any sites 27, knee OA 10, hip OA 26) (Table S2). 

### Statistical analysis

#### MR analysis

The causal relationship between each exposure and each outcome was assessed using the inverse variance weighted (IVW) approach with a fix effect model. We excluded instrumental variables (IVs) that were substantially linked with outcome. The IVW approach was often regarded as the most trustworthy indicator in MR analysis when there was no sign of directional pleiotropy (*P* for MR-Egger intercept > 0.05) [15]. The IVW method used the log (*OR*)/*β* coefficient of the disease SNP divided by the log (*OR*)/*β* coefficient of the exposed SNP to obtain the Wald estimates for each SNP, and then combined these Wald estimates using a method similar to meta-analysis [16]. When each genetic variation satisfied the IV hypothesis, the IVW method could provide a consistent estimate of the causal effect of exposure on the outcome. Cochran’s *Q* statistics were used to evaluate the IV heterogeneity. In order to further confirm the reliability of MR estimations, we used the MR-Pleiotropy Residual Sum and Outlier techniques (MR-PRESSO), which can identify and eliminate probable pleiotropic IVs and offer the outlier-adjusted estimates, to the IVs for IVW analysis (*P*-value < 0.05). In addition, we used weighted median, simple median, and MR-Egger as complementary analysis methods to test the robustness of the IVW method using random-effect model estimation. Utilizing aggregate data, the weighted median estimate was produced to give protection against ineffective instruments and reliable estimations of causation if at least 50% of the weight originates from IVs [17].

#### Subgroup analysis of age

Considering IVs of TB-BMD are from GWAS analyses of all age groups which may lead to a decrease in the accuracy of MR analyses [9], we did a detailed analysis by age groups. We used the IVW method of the random-effects model to evaluate the causal effects of each exposure and each outcome. TB-BMD data on the basis of age was divided into four stages, which were 15 to 30 (*n* = 4,180), 30 to 45 (*n* = 10,062), 45 to 60 years old (*n* = 18,805), and over 60 years old (*n* = 22,504), respectively [9]. MR analysis method was used to estimate the correlation between OA and the corresponding phase TB-BMD.

#### Gene ontology enrichment analysis

The gene ontology (GO) [18] which involves biological process, molecular function, and cellular component were analyzed to further validate whether the potential targets were indeed related to OA. We connected the causative BMD lead SNPs identified in several OA symptoms to the adjacent genes. After using the Benjamini–Hochberg method for adjustment [19], *P* < 0.05 was used as the significance cutoff in our study.

#### Expression quantitative trait locus analysis

We tested the relationships between OA and OP features using a gene-based strategy and extrapolated GTEx V746 expression levels for the musculoskeletal tissues. The elastic net model is used by MetaXcan [20] to impute the cis-genetic component of expression into a much bigger set for a collection of reference participants for whom both gene expression and genetic variation have been assessed. It then did a transcriptome-wide association study to identify meaningful expression-trait connections and corroborated the imputed gene expression to the trait of interest. With 20,000 genes spread over 48 tissues, we employed a conservative Bonferroni correction to account for the gene-tissue pairings, which resulted in a significance threshold of 5.20 × 10^−8^. We calculated the likelihood of each GWAS and expression quantitative trait locus (eQTL) signal colocalizing in each significant MetaXcan results using Coloc47 to lessen the impact of LD confounding on the MetaXcan results when various causal SNPs were affecting expression levels and phenotypic traits in a GWAS [21].

R 3.5.3 and STATA 14.0 were used to conduct all statistical analyses. Unless otherwise stated, statistical significance was defined as *P* < 0.05. Because the objective of our study was the causal relationship between different types of BMD and OA, we did not apply a multiple testing correction to the reported *P*. GO enrichment analysis was performed by websites tool “FUMA” at http://fuma.ctglab.nl.

## Results

### Stage 1: forward MR analysis of the effects of BMD on OA

By applying a two-sample MR approach, we first investigated the causal associations of low BMD on OA. We identified 47 (FN-BMD), 47 (LS-BMD), and 43 (TB-BMD) IVs from GWAS that met genome-wide significance level (*P* < 5 × 10^−8^) based on the elimination of certain missing data (*r*^2^ < 0.001). The heterogeneity test showed no significant heterogeneity among selected IVs (Q^_^*P* > 0.05, Table 1), indicating our MR results were not biased by heterogeneity or horizontal pleiotropy. Table S1 shows the effect sizes of a few IVs.

**Table 1:** Mendelian randomization estimates for bone mineral density on osteoarthritis

Exposure	Outcome	No. of IVs	MR results		Heterogeneity tests	
			**Method**	***β* (95% CI)**	***P*-value**	**Cochran’s *Q* (*P*)**
**FN-BMD**	**OA at any sites**	**47**	**Simple median**	0.032 (− 0.031, 0.095)	0.317	30.869 (0.473)
			**Weighted median**	0.032 (− 0.032, 0.096)	0.324	
			**IVW**	0.029 (− 0.014, 0.071)	0.183	
			**MR-Egger**	0.005 (− 0.145, 0.154)	0.952	
			**MR-Egger intercept**	0.001 (− 0.006, 0.009)	0.739	
	**Knee OA**		**Simple median**	0.097 (− 0.002, 0.196)	0.054	22.974 (0.904)
			**Weighted median**	0.100 (0.003, 0.196)	**0.043**	
			**IVW**	0.117 (0.049, 0.184)	**0.001**	
			**MR-Egger**	0.169 (− 0.061, 0.399)	0.150	
			**MR-Egger intercept**	− 0.003 (− 0.014, 0.009)	0.641	
	**Hip OA**		**Simple median**	0.169 (0.048, 0.291)	**0.006**	27.175 (0.825)
			**Weighted median**	0.136 (0.017, 0.255)	**0.025**	
			**IVW**	0.105 (0.023, 0.188)	**0.012**	
			**MR-Egger**	− 0.140 (− 0.420, 0.139)	0.325	
			**MR-Egger intercept**	0.013 (− 0.001, 0.027)	0.071	
**LS-BMD**	**OA at any sites**	**47**	**Simple median**	0.088 (0.034, 0.141)	**0.001**	24.319 (0.665)
			**Weighted median**	0.065 (0.013, 0.117)	**0.014**	
			**IVW**	0.048 (0.011, 0.085)	**0.010**	
			**MR-Egger**	0.013 (− 0.120, 0.146)	0.852	
			**MR-Egger intercept**	0.002 (− 0.006, 0.010)	0.586	
	**Knee OA**		**Simple median**	0.085 (0.005, 0.165)	**0.037**	22.078 (0.956)
			**Weighted median**	0.116 (0.038, 0.195)	**0.003**	
			**IVW**	0.101 (0.045, 0.156)	**0.001**	
			**MR-Egger**	0.164 (− 0.047, 0.376)	0.127	
			**MR-Egger intercept**	− 0.004 (− 0.017, 0.009)	0.541	
	**Hip OA**		**Simple median**	0.174 (0.072, 0.276)	**0.001**	17.112 (0.960)
			**Weighted median**	0.138 (0.037, 0.239)	**0.008**	
			**IVW**	0.150 (0.077, 0.224)	**0.001**	
			**MR-Egger**	− 0.025 (− 0.264, 0.214)	0.839	
			**MR-Egger intercept**	0.011 (− 0.003, 0.026)	0.131	
**TB-BMD**	**OA at any sites**	**43**	**Simple median**	0.049 (− 0.013, 0.112)	0.122	37.783 (0.127)
			**Weighted median**	0.054 (− 0.008, 0.117)	0.086	
			**IVW**	0.025 (− 0.020, 0.070)	0.270	
			**MR-Egger**	0.078 (− 0.038, 0.195)	0.189	
			**MR-Egger intercept**	− 0.003 (− 0.007, 0.003)	0.335	
	**Knee OA**		**Simple median**	0.028 (− 0.066, 0.123)	0.553	30.115 (0.408)
			**Weighted median**	0.029 (− 0.067, 0.125)	0.554	
			**IVW**	0.043 (− 0.023, 0.109)	0.199	
			**MR-Egger**	0.050 (− 0.126, 0.226)	0.578	
			**MR-Egger intercept**	0.001 (− 0.010, 0.009)	0.937	
	**Hip OA**		**Simple median**	0.117 (− 0.010, 0.244)	0.071	30.700 (0.532)
			**Weighted median**	0.121 (− 0.008, 0.251)	0.066	
			**IVW**	0.092 (0.010, 0.174)	**0.027**	
			**MR-Egger**	0.148 (− 0.069, 0.365)	0.182	
			**MR-Egger intercept**	− 0.003 (− 0.014, 0.008)	0.589	

*BMD* bone mineral density, *OA* osteoarthritis, *FN* femoral neck, *LS* lumbar spine, *TB* total body, *IVs* instrumental variables, *IVW* inverse variance weighted

The IVW method showed genetically predicted low FN-BMD were positively associated with knee OA (*β* = 0.117, 95% CI: 0.049–0.184) and hip OA (*β* = 0.105, 95% CI: 0.023–0.188), but not with OA at any sites (*β* = 0.029, 95% CI: − 0.014–0.071). Genetically predicted low LS-BMD was positively associated with OA at any sites (*β* = 0.048, 95% CI: 0.011–0.085), knee OA (*β* = 0.101, 95% CI: 0.045–0.156) and hip OA (*β* = 0.150, 95% CI: 0.077–0.224). Genetically predicted low TB-BMD was positively associated with hip OA (*β* = 0.092, 95% CI: 0.010–0.174), but not with OA at any sites (*β* = 0.025, 95% CI: − 0.020–0.070), or knee OA (*β* = 0.043, 95% CI: − 0.023–0.109). Causal estimates from the weighted median and simple median revealed similar findings of FN-BMD on hip OA and OA at any sites: LS-BMD on knee OA, hip OA, and OA at any sites (*β* ranged from 0.065 to 0.174). The weighted median showed causal association between low FN-BMD and knee OA (*β* = 0.100, 95% CI: 0.003–0.196). MR-Egger showed no causal effect of BMD on OA phenotypes. Detailed data are shown in Table 1. To test the authenticity of these analyses, we used the summary data on OA released in 2021 [11] for a secondary calculation, and the results were largely consistent (Table S2).

### Stage 2: reverse MR analysis of the effects of OA on BMD

Using OA-associated SNPs as IVs, we further investigated the causative relationships between OA and low BMD in the reversal direction. We obtained 26 (OA at any sites), 10 (knee OA), and 26 (hip OA) IVs without effects of LD-independent (*r*^2^ < 0.001), reaching *P* < 1 × 10^−5^ from GWAS. When examining the causal relationship of OA at any sites on FN-BMD and TB-BMD, we deleted one of the SNPs (rs528981060) because of its significant heterogeneity on outcomes. The heterogeneity test showed no significant heterogeneity (Q^_^*P* > 0.05, Table 2) in selected IVs of OA on low BMD, demonstrating that neither horizontal pleiotropy nor heterogeneity affected our MR findings. The negative control analysis showed that OA was not associated with selected IVs, suggesting that the selected IVs for exposures in this study were appropriate (Table S3).

**Table 2:** Mendelian randomization estimates for osteoarthritis on bone mineral density

Exposure	Outcome	No. of IVs	MR results			Heterogeneity tests
			**Method**	***β* (95% CI)**	***P*-value**	**Cochran’s *Q* (*P*)**
**OA at any sites**	**FN-BMD**	^a^	**Simple median**	0.065 (− 0.123, 0.133)	0.940	21.201 (0.508)
			**Weighted median**	0.063 (− 0.110, 0.137)	0.831	
			**IVW**	0.044 (− 0.095, 0.078)	0.848	
			**MR-Egger**	0.149 (− 0.115, 0.470)	0.235	
			**MR-Egger intercept**	− 0.008 (− 0.020, 0.004)	0.192	
	**LS-BMD**	**27**	**Simple median**	0.079 (− 0.133, 0.175)	0.790	33.890 (0.067)
			**Weighted median**	0.078 (− 0.132, 0.176)	0.779	
			**IVW**	0.061 (− 0.079, 0.162)	0.449	
			**MR-Egger**	0.181 (− 0.074, 0.636)	0.121	
			**MR-Egger intercept**	− 0.010 (− 0.025, 0.004)	0.161	
	**TB-BMD**	^a^	**Simple median**	0.051 (− 0.100, 0.100)	1.000	21.294 (0.503)
			**Weighted median**	0.049 (− 0.078, 0.113)	0.722	
			**IVW**	0.035 (− 0.075, 0.061)	0.837	
			**MR-Egger**	0.129 (− 0.402, 0.104)	0.249	
			**MR-Egger intercept**	0.006 (− 0.004, 0.016)	0.254	
**Knee OA**	**FN-BMD**	**10**	**Simple median**	0.057 (− 0.170, 0.054)	0.310	4.883 (0.674)
			**Weighted median**	0.056 (− 0.200, 0.019)	0.104	
			**IVW**	0.043 (− 0.142, 0.027)	0.185	
			**MR-Egger**	0.225 (− 0.661, 0.222)	0.330	
			**MR-Egger intercept**	0.011 (− 0.018, 0.040)	0.464	
	**LS-BMD**		**Simple median**	0.075 (− 0.028, 0.268)	0.112	5.986 (0.541)
			**Weighted median**	0.077 (− 0.036, 0.265)	0.137	
			**IVW**	0.056 (− 0.028, 0.192)	0.145	
			**MR-Egger**	0.791 (− 1.328, 1.771)	0.780	
			**MR-Egger intercept**	− 0.009 (− 0.104, 0.087)	0.860	
	**TB-BMD**		**Simple median**	0.045 (− 0.059, 0.116)	0.524	12.840 (0.076)
			**Weighted median**	0.046 (− 0.066, 0.114)	0.602	
			**IVW**	0.045 (− 0.077, 0.099)	0.810	
			**MR-Egger**	0.261 (− 0.633, 0.388)	0.639	
			**MR-Egger intercept**	0.009 (− 0.025, 0.044)	0.603	
**Hip OA**	**FN-BMD**	**26**	**Simple median**	0.032 (− 0.044, 0.083)	0.538	17.608 (0.482)
			**Weighted median**	0.031 (− 0.045, 0.078)	0.593	
			**IVW**	0.022 (− 0.043, 0.044)	0.981	
			**MR-Egger**	0.111 (− 0.231, 0.205)	0.906	
			**MR-Egger intercept**	0.001 (− 0.018, 0.021)	0.900	
	**LS-BMD**		**Simple median**	0.035 (− 0.046, 0.090)	0.530	12.702 (0.854)
			**Weighted median**	0.034 (− 0.047, 0.087)	0.561	
			**IVW**	0.025 (− 0.024, 0.074)	0.317	
			**MR-Egger**	0.093 (− 0.271, 0.095)	0.345	
			**MR-Egger intercept**	0.011 (− 0.006, 0.029)	0.208	
	**TB-BMD**		**Simple median**	0.025 (− 0.073, 0.025)	0.333	20.549 (0.303)
			**Weighted median**	0.024 (− 0.072, 0.023)	0.316	
			**IVW**	0.018 (− 0.052, 0.018)	0.353	
			**MR-Egger**	0.073 (− 0.183, 0.103)	0.582	
			**MR-Egger intercept**	0.002 (− 0.011, 0.016)	0.739	

*BMD* bone mineral density, *OA* osteoarthritis, *FN* femoral neck, *LS* lumbar spine, *TB* total body, *IVs* instrumental variables, *IVW* inverse variance weighted

^a^IVs are 26, SNP (rs528981060) overlaps the outcome

The IVW method showed that genetically predicted three OA exposures were not associated with three BMD outcomes (*β* ranged from 0.018 to 0.061). The estimates from MR-Egger, simple median, and weighted median were largely similar (*β* ranged from 0.025 to 0.181) (Table 2).

### Subgroup analysis by age

Subgroup analyses by age were showed in Table S4. In those with 30–45 years of age, TB-BMD only had a strong causal effect on OA at any sites (*P* = 0.017) and hip OA (*P* = 0.021). TB-BMD had a strong causal effect on all the OA phenotypes in participants between 45 and 60 years of age (OA at any sites, *P* = 0.001; knee OA, *P* = 0.001; hip OA, *P* = 0.009). In those above 60 years of age, TB-BMD also had a strong causal effect on OA at any sites, knee OA and hip OA. However, we did not find valid data for MR estimates (data not shown) in participants below 30 years of age. Due to the absence of FN-BMD and LS-BMD subgroup data in different age groups, we were unable to complete the relevant MR analysis.

### GO enrichment analysis

**Fig. 2:**
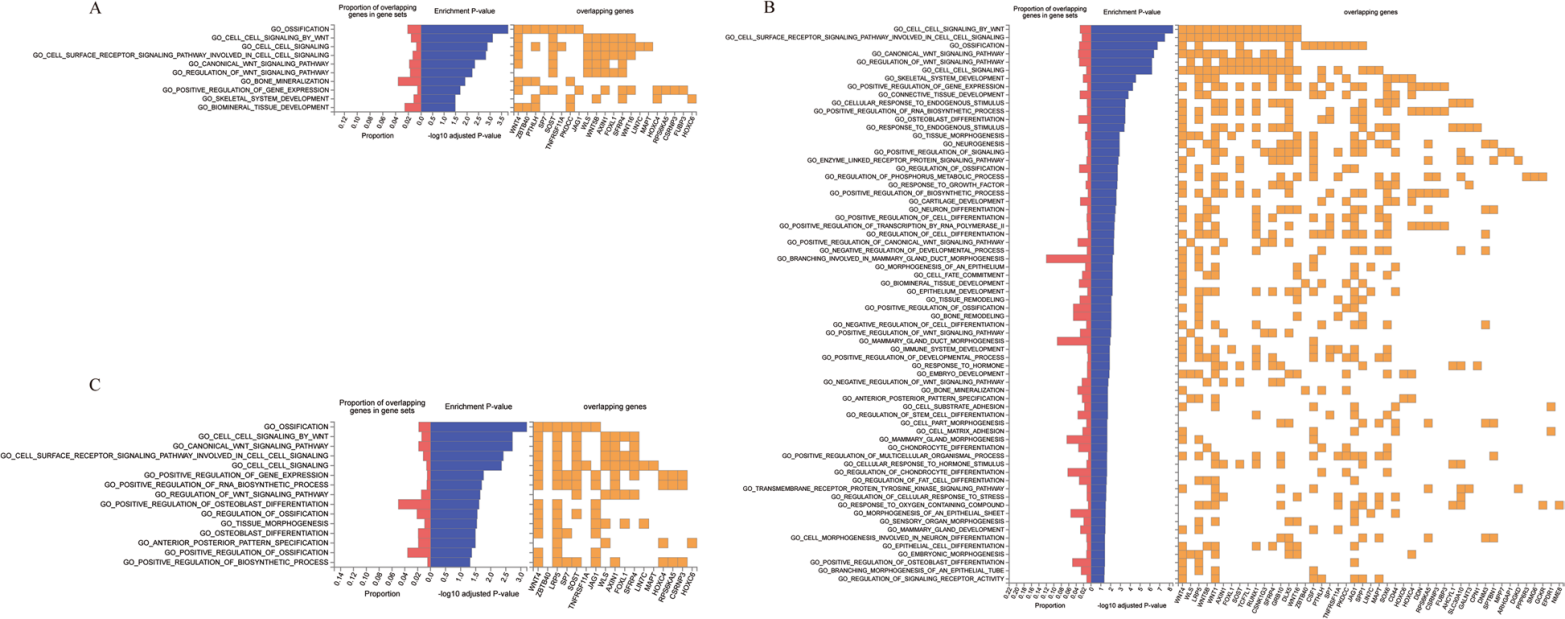
Gene Ontology enrichment analysis of the casual effect of Bone Mineral Density on Osteoarthritis. **A**, Gene Ontology enrichment analysis of the casual effect of Bone Mineral Density on knee Osteoarthritis. **B**, Gene Ontology enrichment analysis of the casual effect of Bone Mineral Density on Hip Osteoarthritis. **C**, Gene Ontology enrichment analysis of the casual effect of Bone Mineral Density on Osteoarthritis at any sites

The findings of the GO enrichment study are shown in Fig. 2. BMD on different OA sites revealed high enrichment in a number of important regulatory pathways according to GO enrichment analyses. For knee OA, 10 GO biological processes were observed to be involved, such as ossification, cell signaling by WNT, and bone mineralization (Fig. 2A). For hip OA, 68 GO biological processes were observed to be involved and the most significant ones were presented in Fig. 2, such as cell signaling by WNT, cell surface receptor signaling pathway, ossification, skeletal system development and neurogenesis (Fig. 2B). For OA at any sites, 15 GO biological processes were observed to be involved, such as regulation of gene expression, RNA biosynthetic process, tissue morphogenesis and osteoblast (Fig. 2C).

### eQTL co-location analysis

The eQTL colocation analysis showed that four BMD susceptibility genes (*ANAPC1*, *GALNT3*, *LIN7C*, and *DDN*) had variants co-located on musculoskeletal tissues (Table 3). We found that gene *GALNT3* was widely presented in all three types of BMD, whose variants co-located on LS-BMD were the most prominent (rs1346004, rs11680288). Among all susceptibility genes with variants co-located discovered, the effect of gene *DDN* was the largest (*P* < 0.001). We also found four hip OA susceptibility genes (*LTBP3*, *MLXIP*, *SMAD3*, and *MAPT*) that had variants co-located on musculoskeletal tissues. For hip OA co-located variants, the effect of gene *SMAD3* was the largest (*P* < 0.001), followed by *MAPT*, *LTBP3*, and *MLXIP* (all *P* < 0.001). No genes had variants co-located on knee OA or OA at any sites.

**Table 3:** Significant eQTL signals for osteoporosis and osteoarthritis from musculoskeletal tissues based on the GTEx data

Direction	Ensemble ID	Gene Symbol	CHR	POS	SNP ID	*P*-value	Normalized effect size	Tissue
**FN-BMD**	ENSG00000153107.11	ANAPC1	2	112500035	rs17040773	2.80 × 10^−07^	0.190	Muscle-Skeletal
	ENSG00000115339.13	GALNT3	2	166601046	rs1346004	1.20 × 10^−10^	− 0.280	
**LS-BMD**	ENSG00000148943.11	LIN7C	11	27505677	rs10835187	0.00015	− 0.100	
	ENSG00000115339.13	GALNT3	2	166601046	rs1346004	1.20 × 10^−10^	− 0.280	
	ENSG00000115339.13	GALNT3	2	166603281	rs11680288	1.00 × 10^−10^	− 0.280	
**TB-BMD**	ENSG00000181418.7	DDN	12	49385679	rs10875906	6.60 × 10^−17^	− 0.310	
	ENSG00000115339.13	GALNT3	2	166577489	rs7586085	2.30 × 10^−10^	− 0.280	
**Hip OA**	ENSG00000168056.15	LTBP3	11	65323725	rs10896015	1.50 × 10^−11^	0.190	
	ENSG00000175727.13	MLXIP	12	122606837	rs11059094	9.60 × 10^−07^	− 0.100	
	ENSG00000166949.15	SMAD3	15	67370506	rs12901372	5.60 × 10^−18^	0.270	
	ENSG00000264589.2	MAPT	17	44038785	rs62063281	3.90 × 10^−12^	− 0.350	

*eQTL* expression quantitative trait locus, *BMD* bone mineral density, *OA* osteoarthritis, *CHR* chromosome, *POS* position, *P*-value: the statistical analysis of enrichment score effect size, which is used to characterize the credibility of enrichment results

## Discussion

Using the bi-directional two-sample MR method, we found evidence for a causal association of low BMD on OA, and the association was more prominent in people over 45 years old. No clear evidence of a causal relationship from OA to BMD was found. The eQTL co-location analysis revealed that four BMD susceptibility genes (*ANAPC1*, *GALNT3*, *LIN7C*, and *DDN*) and four hip OA susceptibility genes (*LTBP3, MLXIP*, *SMAD3*, and *MAPT*) had variants co-located on musculoskeletal tissues, suggesting these genes may play roles in regulating the development of OP and OA.

Previous studies reported that OP may be one of the etiologies to promote OA development. A meta-analysis showed that the worldwide incidence of OP increased with aging, and could promote OA through a number of mechanisms [22]. For example, a prospective study showed older participants with radiographic hip and knee OA had higher total hip bone loss over 2.6 years [23]. Developing OP has been shown to aggravate cartilage lesions in an experimental model of OA in rabbits [24]. Similar findings were made by Bellido M. et al., who discovered that the fragility and poor quality of subchondral bone may also increase cartilage injury in addition to promoting bone remodeling [25]. An increased rate of bone turnover has been shown in animal models of early OA [26]. In cadaver specimens of people with early OA, the elastic modulus of trabecular subchondral bone from proximal tibiae decreased despite an increase in bone volume [27]. This local bone softening may accompany a drop in BMD, which may be the result of inadequate mineralization brought on by increased bone remodeling [27]. In summary, our findings are generally consistent with the above observational studies that low BMD may be associated with an increased risk of OA. In contrast, there were some observational studies showing that higher femoral neck and total body BMD were associated with an increased risk of incident OA [28]. A cross-sectional and longitudinal study reported a positive correlation between subchondral BMD and OA severity in patients [29]. In general, these studies indicated the detrimental effect of higher BMD on OA development.

Regarding the possible causal relationship from OA to OP, results varied among studies. The MR Study by Hartley et al. suggested that a reduction in BMD would increase the incidence of OA, while Lin et al. suggested that a reduction in bone mineral density had a protective effect on OA [30, 31]. The possible explanations for the discrepancies include (1) different sources of exposure and differences in the final selection of SNPs, (2) although we tried to minimize phenotypic heterogeneity, it could not disappear, (3) different methods for assessing OA and/or OP [27], and (4) lack of adjustment for essential confounders such as age, gender, weight, and/or BMI [32]. The protective effect of high BMD on OA may be through its effect on reducing the risk of joint space loss [5], and the mechanism of OP causing OA could be the bone loss in the subchondral bone resulting in the collapse of the articular surface and leading to uneven stress on the articular cartilage, which leads to secondary osteophyte proliferation and cartilage damage [33]. It is worth noting that Lin et al. [31] only analyzed the causal relationship from OP to OA, and the summary data of exposure and outcome in their study were both from UK Biobank, which may cause bias in the results. Based on two separate OA summary datasets, our findings largely agreed with Hartley et al.’s [30], indicating that improving BMD might help to prevent OA from a genetic standpoint. Kindly noted the previously study assessed BMD by heel ultrasound as the only exposure, while our study used dual-energy X-ray assessed FN-BMD, LS-BMD, and TB-BMD as the exposures.

In our study, we found that the causal effect of FN-BMD on knee OA was stronger than that of hip OA, while LS-BMD had a strong causal effect on OA at any sites, knee OA and hip OA. Hackinger et al. [34] discovered a genetic link between LS-BMD (but not FN) and OA, which is consistent with common biological pathways causing both BMD and OA. On the other hand, TB-BMD only had a weak causal effect on hip OA, possibly because of the special trabecular structure of the hip [35]. Moreover, although we used two databases for verification, we cannot rule out the possibility of false positives. In the reverse MR analyses, no evidence for a causal effect of OA on BMD was found. This could be due to the influence of the genetic variation on the result is not entirely mediated by the binary exposure (OA definition), power estimations are probably conservative in this case. However, estimating approaches for a binary exposure call for strong assumptions that are unlikely to be physiologically tenable in typical MR situations, making it difficult to perform tests for causal effects without employing exposure information [36].

GO enrichment analysis found that a large number of GO biologic processes play key roles in the potential causal relationship from low BMD to OA, such as ossification [37], cell surface receptor signaling pathway [38], and cell signaling by *WNT* [39]. Using OP mice mode, Wu et al. discovered that nine cellular elements, including the cytosol, nucleus, cytoplasm, neuronal cell body, protein complex, caveola, and endoplasmic reticulum, were involved in the anti-OP effects on OA [37]. In addition, our GO analysis revealed that the primary ways in which OA affected OP were via inflammatory response, aging, responses to estradiol, glucocorticoids, and hydrogen peroxide [37]. The study by Guan et al. suggested that XianLing GuBao Capsule (*XLGB*) might treat OP through the *PI3K/AKT* and *MAPK* pathways [38]. Similarly, Xiao et al. found that *PI3K/AKT/NF-κB* and *MAPK* pathways would be the potential mechanism for *XLGB* in the treatment of OA [39]. Additionally, aberrant bone remodeling and reduced mineralization have been linked to abnormal OPG and RANKL expression in OA osteoblasts [40]. *WNT* protein is considered as a risk factor for the onset and development of OA. There are two primary types of *WNT* signaling pathways: canonical and non-canonical. Of these, the canonical *WNT* signaling pathway promotes osteogenesis. Osteocytes generate the inhibitor of this process, sclerostin, which prevents osteogenesis [41]. Since we substituted BMD SNPs for OP, it is reasonable to assume that OP also has these biological processes and *WNT* signaling could be potential pathways to be targeted for treating both diseases.

Through eQTL colocation analysis [21], 3 SNPs (rs1346004, rs11680288, and rs7586085) were co-located at gene *GALNT3*. An observational study found that the *GALNT3* gene significantly correlated with OP and the low expression of the *GALNT3* gene can promote the occurrence and deterioration of OP [42]. Our data showed that three newly discovered genes had eQTL effects with OP (*ANAPC1*, *LIN7C*, and *DDN*). In addition, previous reports deemed that the *LIN7C* gene significantly correlates with knee OA [43]. Our study also found four genes with eQTL effect on hip OA (*LTBP3*, *MLXIP*, *SMAD3*, and *MAPT*). The *LTBP3* gene may be involved in the occurrence and development of OA by regulating the *TGF-β* signaling pathway [44]. *SMAD3* gene is the target gene of circ-RNA (Circ0083429), which regulates the mRNA level of *SMAD3* through the sponging of miRNA-346, thus affecting OA. Studies have found that the *SMAD3* gene plays an important role in cartilage bone reconstruction and maintenance, and have confirmed the differential expression of *SMAD3* in intact and degraded knee and hip cartilage [34]. Top-level differentially expressed genes in OA bone, such as those in the *WNT* signaling pathway (*TWIST1*, *IBSP*, *S100A4*, *MMP25*, *RUNX2*, and *CD14*) and *TGF-β/SMAD3* signaling pathway, are known to function in osteoblasts, osteocytes, and osteoclasts (*ADAMTS4*, *ADM*, *MEPE*, *GADD45B*, *COL4A1*, and *FST*) [45]. *MLXIP* and *MAPT* have not been reported to be related to the development of hip OA. Therefore, these newly discovered genes need further confirmation.

The strengths of the current study lie on: First, to minimize possible false-positive results, summary data from two different organizations were used for outcomes, and the latest summary data of OA were also used for verification [10, 11], proving the credibility of our conclusions. Second, four methods of MR analysis on 18 groups of BMD GWAS and OA GWAS summary data were performed, and subgroup analyses by age were performed for TB-BMD. Third, we used GO Enrichment analysis and eQTL to search for potential enrichment genes and to determine whether there are possible co-acting pathways.

Limitations of our study include: First, the majority of the GWAS summary data stem from people of European origin, therefore, we should be cautious when applying our findings to people with other racial and ethnic backgrounds. Second, we evaluated a linear correlation between OP and OA in our MR analysis, but we did not account for the potential of different shapes of association [15]. Third, because summary statistics rather than raw data were used in the analysis, it was not possible to perform subgroup analyses, such as stratified by sex or ethnicity. Last, the results of GO and eQTL were based only on bioinformatic analyses, in vitro and in vivo experiments are needed in the future to validate our findings.

## Conclusions

These findings suggested that OP may be causally linked to an increased risk of OA, indicating that measures to raise BMD may be effective in preventing OA. More research is required to determine the underlying processes via which OP causes OA.

### Supplementary Information

**Additional file 1: Table S1.** Results of two-sample Mendelian randomization analyses of bone mineral density on osteoarthritis. **Table S2.** Mendelian randomization estimates for bone mineral density on osteoarthritis in alternative summary data. **Table S3.** Results of two-sample Mendelian randomization analyses of osteoarthritis on bone mineral density. **Table S4.** Subgroup analysis using Mendelian randomization estimates for bone mineral density on osteoarthritis by age.

## Data Availability

All data comes from public databases.
