# Supplementary material for: Osteoporosis and osteoarthritis: a bi-directional Mendelian randomization study
Source: Arthritis Res Ther. 2023 Dec 13;25:242. doi: 10.1186/s13075-023-03213-5 (PMC10717893; doi:10.1186/s13075-023-03213-5)
Supplement: Supplementary file 1 — Additional file 1: Table S1. Results of two-sample Mendelian randomization analyses of bone mineral density on osteoarthritis. Table S2. Mendelian randomization estimates for bone mineral density on osteoarthritis in alternative summary data. Table S3. Results of two-sample Mendelian randomization analyses of osteoarthritis on bone mineral density. Table S4. Subgroup analysis using Mendelian randomization estimates for bone mineral density on osteoarthritis by age. [file 13075_2023_3213_MOESM1_ESM.pdf]

Additional file 1

Table S1. Results of two-sample Mendelian randomization analyses of bone mineral density on osteoarthritis.

SNP (position)	Effect allele/ Other allele	SNP & OA		SNP & BMD associations					
				OA at any site		Knee OA		Hip OA	
		Beta (SE)	*P*	*Beta* (SE)	*P*	*Beta* (SE)	*P*	*Beta* (SE)	*P*
Femoral neck BMD									
rs752190(1:22490724)	C/A	0.040(0.007)	2.90×10^{-09}	0.022 (-0.007)	0.001	-0.015(0.011)	0.179	-0.030(0.014)	0.028
rs642674(1:22711473)	C/G	0.110(0.007)	7.40×10^{-57}	0.004 (-0.007)	0.628	0.021(0.012)	0.079	0.000(0.015)	0.999
rs12407028(1:68647716)	T/C	0.050(0.005)	3.40×10^{-23}	0.009 (-0.006)	0.127	0.018(0.010)	0.057	0.010(0.012)	0.407
rs2566752(1:68656697)	C/T	0.062(0.008)	3.65×10^{-15}	0.013 (-0.006)	0.025	-0.017(0.010)	0.072	-0.029(0.012)	0.016
rs7084921(10:101813802)	T/C	0.030(0.005)	9.00×10^{-10}	0.004 (-0.006)	0.471	-0.004(0.009)	0.667	0.004(0.012)	0.741
rs1373004(10:54427825)	G/T	0.040(0.007)	1.50×10^{-08}	0.018 (-0.009)	0.048	-0.009(0.015)	0.565	-0.024(0.019)	0.206
rs7108738(11:15710084)	G/T	0.080(0.007)	1.10×10^{-32}	0.008 (-0.007)	0.260	0.035(0.012)	0.005	-0.012(0.015)	0.446
rs163879(11:30951674)	G/T	0.030(0.005)	2.10×10^{-08}	0.026 (-0.006)	0.000	-0.021(0.010)	0.035	-0.028(0.012)	0.025
rs7932354(11:46722221)	T/C	0.050(0.006)	5.10×10^{-18}	0.002 (-0.006)	0.807	0.003(0.010)	0.762	0.008(0.013)	0.554
rs1785493(11:68208345)	T/C	-0.04(0.008)	4.06×10^{-08}	0.008 (-0.006)	0.182	-0.009(0.010)	0.352	-0.031(0.012)	0.012
rs2887571(12:1638171)	G/A	0.030(0.005)	6.50×10^{-09}	0.004 (-0.006)	0.587	0.001(0.011)	0.924	-0.010(0.014)	0.485
rs7953528(12:28017159)	A/T	0.050(0.007)	1.90×10^{-12}	0.005 (-0.007)	0.462	0.022(0.012)	0.077	-0.028(0.015)	0.066
rs2016266(12:53727955)	G/A	0.030(0.005)	3.70×10^{-10}	0.009 (-0.006)	0.147	-0.002(0.010)	0.825	-0.016(0.012)	0.204
rs736825(12:54417576)	C/G	0.040(0.007)	1.10×10^{-09}	0.002 (-0.006)	0.688	-0.013(0.010)	0.172	0.009(0.012)	0.476
rs4759320(12:54433011)	C/G	-0.04(0.009)	3.33×10^{-08}	0.001 (-0.006)	0.857	0.007(0.010)	0.459	-0.009(0.012)	0.484
rs9533090(13:42951449)	C/T	0.050(0.005)	4.90×10^{-23}	0.022 (-0.006)	0.000	-0.040(0.009)	0.000	-0.015(0.012)	0.211

rs1286083(14:91442779)	C/T	0.050(0.006)	2.00×10^{-15}	0.007 (-0.007)	0.342	-0.013(0.012)	0.295	-0.038(0.015)	0.014
rs13336428(16:1532463)	G/A	0.040(0.005)	1.50×10^{-16}	0.012 (-0.006)	0.031	-0.016(0.009)	0.083	-0.040(0.012)	0.001
rs9921222(16:375782)	C/T	0.040(0.006)	5.20×10^{-12}	0.001 (-0.006)	0.820	-0.004(0.009)	0.635	-0.002(0.012)	0.872
rs10794639(16:377367)	G/A	-0.05(0.008)	3.30×10^{-11}	0.001 (-0.006)	0.796	0.005(0.009)	0.615	0.002(0.012)	0.878
rs1566045(16:51021803)	C/T	0.060(0.006)	1.90×10^{-22}	0.003 (-0.007)	0.646	-0.009(0.012)	0.443	-0.005(0.015)	0.765
rs10048146(16:86710660)	A/G	0.050(0.007)	1.00×10^{-14}	0.005 (-0.007)	0.495	-0.029(0.012)	0.013	-0.014(0.015)	0.341
rs7209460(17:2048713)	C/T	-0.05(0.008)	1.35×10^{-09}	0.025 (-0.006)	0.000	0.037(0.010)	0.000	0.014(0.013)	0.279
rs4790881(17:2068932)	A/C	0.050(0.006)	9.80×10^{-19}	0.025 (-0.006)	0.000	0.036(0.010)	0.000	0.015(0.013)	0.230
rs4792909(17:41798824)	T/G	0.040(0.006)	2.00×10^{-11}	0.01 (-0.006)	0.099	0.017(0.010)	0.084	0.018 (0.012)	0.131
rs7217932(17:69949016)	A/G	0.030(0.005)	1.90×10^{-11}	0.021 (-0.006)	0.000	0.046(0.009)	0.000	0.019(0.012)	0.113
rs4796995(18:13708574)	A/G	0.030(0.006)	4.90×10^{-08}	0.000 (-0.006)	0.981	0.002(0.010)	0.826	-0.002(0.012)	0.903
rs17040773(2:112500035)	A/C	0.040(0.007)	1.50×10^{-09}	0.004 (-0.007)	0.535	0.008(0.011)	0.490	-0.031(0.014)	0.027
rs10170839(2:166572906)	C/A	-0.05(0.008)	1.20×10^{-14}	0.012 (-0.006)	0.030	0.020(0.009)	0.029	0.012(0.012)	0.315
rs1346004(2:166601046)	G/A	0.050(0.005)	1.10×10^{-25}	0.012 (-0.006)	0.029	-0.020(0.009)	0.030	-0.013(0.012)	0.276
rs7584262(2:42250549)	T/C	0.040(0.007)	1.30×10^{-09}	0.007 (-0.007)	0.313	-0.023(0.011)	0.042	-0.002(0.014)	0.886
rs3790160(20:10639988)	T/C	0.040(0.006)	3.60×10^{-12}	0.007 (-0.006)	0.230	0.003(0.009)	0.728	0.004(0.012)	0.712
rs1026364(3:113370010)	T/G	0.030(0.005)	4.10×10^{-10}	0.001 (-0.006)	0.819	-0.005(0.010)	0.622	0.004(0.012)	0.736
rs430727(3:41128564)	C/T	0.060(0.006)	4.40×10^{-25}	0.005 (-0.006)	0.378	0.000(0.009)	0.996	-0.026(0.012)	0.029
rs3755955(4:994414)	G/A	0.060(0.008)	1.50×10^{-14}	0.014 (-0.008)	0.070	-0.006(0.013)	0.636	-0.016(0.016)	0.315
rs1366594(5:88376061)	A/C	0.080(0.005)	4.50×10^{-61}	0.016 (-0.006)	0.003	0.035(0.009)	0.000	0.000(0.012)	1.000
rs13194508(6:127144683)	C/T	-0.05(0.009)	1.30×10^{-08}	0.011 (-0.006)	0.073	-0.033(0.011)	0.002	0.020(0.013)	0.143
rs9478217(6:151874122)	A/G	-0.05(0.008)	1.23×10^{-11}	0.007 (-0.006)	0.237	0.001(0.010)	0.932	0.001(0.012)	0.966
rs4869742(6:151907748)	C/T	0.050(0.006)	4.20×10^{-18}	0.002 (-0.006)	0.730	-0.013(0.010)	0.197	-0.003(0.013)	0.842
rs7751941(6:151946658)	G/A	0.040(0.007)	1.60×10^{-09}	0.015 (-0.007)	0.029	-0.023(0.011)	0.044	-0.008(0.014)	0.566
rs9466056(6:21384613)	G/A	0.040(0.006)	2.70×10^{-13}	0.006 (-0.006)	0.265	-0.003(0.010)	0.788	-0.020(0.012)	0.094
rs3779381(7:120966790)	G/A	0.058(0.009)	2.87×10^{-11}	0.007 (-0.006)	0.290	-0.005(0.011)	0.614	-0.014(0.013)	0.296

rs6959212(7:38128326)	C/T	0.040(0.005)	1.20×10^{-13}	0.009 (-0.006)	0.116	-0.008(0.010)	0.398	-0.026(0.012)	0.036
rs4727338(7:96120675)	C/G	0.080(0.006)	8.10×10^{-48}	0.014 (-0.006)	0.015	0.001(0.010)	0.935	0.039(0.012)	0.001
rs4448201(7:96154912)	G/C	-0.06(0.008)	4.37×10^{-16}	0.013 (-0.006)	0.025	-0.001(0.010)	0.896	0.039(0.012)	0.001
rs1485307(8:120007395)	T/C	0.062(0.008)	2.49×10^{-15}	0.006 (-0.006)	0.307	0.015(0.009)	0.116	-0.019(0.012)	0.105
rs7851693(9:133478827)	C/G	0.050(0.005)	3.40×10^{-22}	0.007 (-0.006)	0.263	-0.010(0.010)	0.310	-0.013(0.012)	0.294
Lumber spine BMD									
rs7521902(1:22490724)	C/A	0.050(0.008)	9.70×10^{-11}	-0.021(-0.007)	0.001	-0.015(0.011)	0.179	-0.030(0.014)	0.028
rs6426749(1:22711473)	C/G	0.110(0.007)	7.40×10^{-57}	0.004 (-0.007)	0.628	0.021(0.012)	0.079	0.000(0.015)	0.999
rs12407028(1:68647716)	T/C	0.080(0.006)	3.10×10^{-45}	0.009 (-0.006)	0.127	0.018(0.010)	0.057	0.010(0.012)	0.407
rs2566752(1:68656697)	C/T	0.083(0.009)	1.49×10^{-19}	-0.013 (-0.006)	0.025	-0.017(0.010)	0.072	-0.029(0.012)	0.016
rs3905706(10:28479942)	T/C	0.050(0.006)	2.40×10^{-16}	-0.005 (-0.007)	0.445	-0.012(0.012)	0.312	-0.017(0.015)	0.261
rs1373004(10:54427825)	G/T	0.060(0.009)	1.60×10^{-12}	-0.018 (-0.009)	0.048	-0.009(0.015)	0.565	-0.024(0.019)	0.206
rs10835187(11:27505677)	C/T	0.030(0.006)	4.90×10^{-08}	-0.005 (-0.006)	0.327	-0.019(0.009)	0.046	-0.016(0.012)	0.173
rs163879(11:30951674)	C/T	0.040(0.006)	2.20×10^{-11}	-0.025 (-0.006)	0.000	-0.021(0.010)	0.035	-0.028(0.012)	0.025
rs7932354(11:46722221)	T/C	0.040(0.006)	5.50×10^{-12}	-0.001 (-0.006)	0.807	0.003(0.010)	0.762	0.008(0.013)	0.554
rs2291467(11:68216756)	T/C	-0.07(0.010)	9.64×10^{-14}	-0.008 (-0.006)	0.189	0.000(0.011)	0.969	-0.025(0.013)	0.063
rs2016266(12:53727955)	G/A	0.050(0.005)	3.00×10^{-20}	-0.008 (-0.006)	0.147	-0.002(0.010)	0.825	-0.016(0.012)	0.204
rs894738(12:54417525)	A/G	-0.06(0.009)	2.00×10^{-11}	0.002 (-0.006)	0.676	0.013(0.010)	0.181	-0.008(0.012)	0.520
rs736825(12:54417576)	C/G	0.050(0.006)	7.70×10^{-16}	-0.002 (-0.006)	0.688	-0.013(0.010)	0.172	0.009(0.012)	0.476
rs9533090(13:42951449)	C/T	0.100(0.006)	4.80×10^{-68}	-0.022 (-0.006)	0.000	-0.040(0.009)	0.000	-0.015(0.012)	0.211
rs9533094(13:42965837)	G/A	-0.08(0.009)	2.80×10^{-20}	0.021 (-0.006)	0.000	0.041(0.009)	0.000	0.015(0.012)	0.214
rs1286083(14:91442779)	C/T	0.050(0.007)	1.80×10^{-14}	-0.007 (-0.007)	0.342	-0.013(0.012)	0.295	-0.038(0.015)	0.014
rs13336428(16:1532463)	G/A	0.040(0.005)	1.70×10^{-13}	-0.012 (-0.006)	0.031	-0.016(0.009)	0.083	-0.040(0.012)	0.001
rs9921222(16:375782)	T/C	-0.05(0.009)	3.16×10^{-09}	-0.001 (-0.006)	0.820	-0.004(0.009)	0.635	-0.002(0.012)	0.872
rs10048146(16:86710660)	A/G	0.050(0.008)	3.10×10^{-11}	-0.005 (-0.007)	0.495	-0.029(0.012)	0.013	-0.014(0.015)	0.341

rs4790881(17:2068932)	A/C	0.030(0.005)	3.40×10^{-09}	0.025 (-0.006)	0.000	0.036(0.010)	0.000	0.015(0.013)	0.230
rs4792909(17:41798824)	T/G	0.040(0.007)	9.40×10^{-10}	0.01 (-0.006)	0.099	0.017(0.010)	0.084	0.018(0.012)	0.131
rs1864325(17:43977827)	C/T	0.040(0.006)	4.90×10^{-11}	0.021 (-0.007)	0.001	-0.008(0.011)	0.481	0.091(0.014)	0.000
rs884205(18:60054857)	A/C	-0.06(0.010)	2.77×10^{-09}	-0.017 (-0.006)	0.006	-0.037(0.011)	0.001	-0.032(0.014)	0.018
rs1878526(2:119038598)	A/G	0.040(0.006)	1.20×10^{-10}	-0.02 (-0.007)	0.002	0.003(0.011)	0.816	-0.003(0.014)	0.853
rs1346004(2:166601046)	G/A	0.060(0.005)	3.90×10^{-30}	-0.012 (-0.006)	0.029	-0.020(0.009)	0.030	-0.013(0.012)	0.276
rs11680288(2:166603281)	G/A	0.054(0.009)	3.12×10^{-09}	-0.011 (-0.006)	0.042	-0.019(0.009)	0.047	-0.011(0.012)	0.348
rs4233949(2:54659707)	C/G	0.050(0.006)	2.30×10^{-18}	-0.001 (-0.006)	0.918	-0.003(0.010)	0.770	0.023(0.012)	0.056
rs3790160(20:10639988)	T/C	0.050(0.006	3.10×10^{-19}	-0.007 (-0.006)	0.230	0.003(0.009)	0.728	0.004(0.012)	0.712
rs2235811(20:10644158)	G/A	-0.05(0.009)	4.66×10^{-09}	-0.006 (-0.006)	0.283	0.003(0.009)	0.736	0.005(0.012)	0.701
rs13046645(21:36818136)	A/T	-0.05(0.010)	2.92×10^{-08}	-0.006 (-0.006)	0.301	-0.009(0.010)	0.400	-0.033(0.013)	0.011
rs344081(3:156555984)	T/C	0.060(0.009)	4.50×10^{-12}	0.018 (-0.008)	0.036	0.016(0.014)	0.274	0.041(0.018)	0.020
rs401680(3:41127094)	A/T	-0.05(0.009)	3.70×10^{-10}	-0.005 (-0.006)	0.368	0.000(0.009)	0.978	-0.026(0.012)	0.026
rs430727(3:41128564)	C/T	0.050(0.006)	1.50×10^{-18}	-0.005 (-0.006)	0.378	0.000(0.009)	0.996	-0.026(0.012)	0.029
rs3755955(4:994414)	G/A	0.060(0.008)	5.20×10^{-15}	-0.014 (-0.008)	0.070	-0.006(0.013)	0.636	-0.016(0.016)	0.315
rs4869742(6:151907748)	C/T	0.080(0.006)	4.00×10^{-35}	0.002 (-0.006)	0.730	-0.013(0.010)	0.197	-0.003(0.013)	0.842
rs1023940(6:151932778)	A/G	0.065(0.009)	6.47×10^{-13}	0.006 (-0.006)	0.284	0.014(0.009)	0.147	0.006(0.012)	0.590
rs7751941(6:151946658)	G/A	0.080(0.008)	2.00×10^{-24}	-0.014 (-0.007)	0.029	-0.023(0.011)	0.044	-0.008(0.014)	0.566
rs9466056(6:21384613)	G/A	0.030(0.006)	3.60×10^{-08}	-0.006 (-0.006)	0.265	-0.003(0.010)	0.788	-0.020(0.012)	0.094
rs11755164(6:44639184)	C/T	0.040(0.006)	5.60×10^{-11}	0.027 (-0.006)	0.000	0.020(0.009)	0.031	0.067(0.012)	0.000
rs13245690(7:120785064)	A/G	0.050(0.008)	1.70×10^{-11}	-0.003 (-0.006)	0.558	0.001(0.010)	0.957	0.016(0.012)	0.183
rs7807953(7:121000718)	T/C	0.075(0.010)	4.11×10^{-14}	0.007 (-0.006)	0.275	0.006(0.010)	0.575	0.015(0.013)	0.258
rs10226308(7:37938422)	G/A	0.060(0.008)	6.40×10^{-13}	-0.004 (-0.007)	0.577	-0.004(0.012)	0.731	0.002(0.015)	0.912
rs1357651(7:38097862)	T/G	-0.06(0.009)	3.75×10^{-13}	-0.01 (-0.006)	0.083	-0.010(0.010)	0.321	-0.025(0.012)	0.045
rs6959212(7:38128326)	C/T	0.040(0.005)	1.20×10^{-13}	-0.009 (-0.006)	0.116	-0.008(0.010)	0.398	-0.026(0.012)	0.036
rs4727338(7:96120675)	C/G	0.070(0.006)	2.10×10^{-35}	0.014 (-0.006)	0.015	0.001(0.010)	0.935	0.039(0.012)	0.001

rs6965122(7:96133319)	G/A	-0.06(0.009)	7.40×10^{-11}	0.014 (-0.006)	0.021	-0.001(0.010)	0.886	0.040(0.012)	0.001
rs2220189(8:120007708)	C/G	0.083(0.009)	4.25×10^{-20}	-0.001 (-0.006)	0.881	0.011(0.009)	0.229	-0.024(0.012)	0.042
Total Body BMD									
rs7548588(1:110475971)	T/C	-0.037(0.006)	2.21×10^{-10}	0.012 (-0.006)	0.033	0.020(0.010)	0.037	0.016(0.012)	0.185
rs633995(1:172186729)	A/G	0.035(0.006)	1.61×10^{-09}	0.015 (-0.006)	0.011	0.027(0.010)	0.005	-0.009(0.012)	0.451
rs61837366(1:220038825)	T/C	0.042(0.007)	3.07×10^{-09}	0.003 (-0.006)	0.685	0.002(0.011)	0.825	0.002(0.014)	0.886
rs1078826(10:124015986)	A/G	-0.034(0.006)	2.61×10^{-09}	-0.009 (-0.006)	0.109	-0.017(0.009)	0.067	-0.036(0.012)	0.002
rs725670(11:121913230)	A/G	-0.032(0.006)	3.61×10^{-08}	0.011 (-0.006)	0.059	0.015(0.010)	0.120	0.022(0.012)	0.071
rs35199438(11:16630779)	T/G	-0.049(0.006)	2.36×10^{-15}	-0.008 (-0.006)	0.183	-0.008(0.010)	0.453	-0.011(0.013)	0.389
rs7105860(11:27306364)	C/G	-0.047(0.006)	2.36×10^{-15}	0.003 (-0.006)	0.604	0.010(0.010)	0.314	0.003(0.012)	0.840
rs2553773(11:35083633)	C/G	-0.037(0.006)	1.49×10^{-10}	0.004 (-0.006)	0.439	0.010(0.009)	0.290	-0.004(0.012)	0.724
rs11228240(11:68218290)	T/C	-0.083(0.007)	1.72×10^{-35}	-0.007 (-0.006)	0.223	-0.002(0.010)	0.857	-0.023(0.013)	0.079
rs1037011(12:107302778)	T/C	-0.040(0.006)	1.54×10^{-12}	-0.019 (-0.006)	0.000	-0.018(0.009)	0.060	-0.029(0.012)	0.015
rs10875906(12:49385679)	T/C	0.051(0.007)	1.85×10^{-13}	0.011 (-0.006)	0.077	0.002(0.011)	0.861	0.042(0.013)	0.001
rs10777212(12:90334829)	T/G	0.045(0.006)	5.05×10^{-14}	0.016 (-0.006)	0.005	0.020(0.010)	0.039	0.033(0.012)	0.007
rs3743347(15:67547301)	A/C	0.052(0.007)	1.75×10^{-14}	-0.017 (-0.007)	0.011	-0.008(0.011)	0.497	0.015(0.014)	0.270
rs8047501(16:392318)	A/G	0.052(0.006)	1.13×10^{-18}	-0.001 (-0.006)	0.841	0.004(0.009)	0.682	0.006(0.012)	0.624
rs8070128(17:17804725)	T/C	-0.039(0.006)	1.98×10^{-11}	0.008 (-0.006)	0.177	0.007(0.010)	0.486	-0.004(0.012)	0.742
rs2873195(17:2064702)	A/T	-0.041(0.006)	4.31×10^{-11}	-0.025 (-0.006)	0.000	-0.036(0.011)	0.001	-0.013(0.014)	0.332
rs9972944(17:63771079)	A/G	0.036(0.006)	6.87×10^{-10}	0.000 (-0.006)	0.941	0.017(0.010)	0.101	-0.014(0.013)	0.288
rs7586085(2:166577489)	A/G	0.053(0.006)	8.64×10^{-21}	0.012 (-0.006)	0.035	0.020(0.009)	0.031	0.012(0.012)	0.302
rs838721(2:234303405)	A/G	-0.031(0.006)	4.48×10^{-08}	-0.021 (-0.006)	0.000	-0.021(0.009)	0.027	-0.015(0.012)	0.214
rs780096(2:27741072)	C/G	-0.031(0.006)	4.58×10^{-08}	-0.006 (-0.006)	0.277	-0.022(0.009)	0.021	-0.025(0.012)	0.035
rs11904127(2:85484818)	A/G	-0.032(0.006)	1.18×10^{-08}	-0.007 (-0.006)	0.216	0.015(0.009)	0.111	-0.031(0.012)	0.009
rs6040063(20:10640877)	A/G	0.036(0.006)	1.78×10^{-10}	-0.006 (-0.006)	0.253	0.003(0.009)	0.717	0.005(0.012)	0.675

rs6029130(20:39103882)	T/C	0.035(0.006)	3.50×10^{-08}	-0.007 (-0.006)	0.236	-0.006 (-0.011)	0.576	-0.002(0.013)	0.902
rs1452102(21:28773868)	T/G	-0.035(0.006)	1.74×10^{-09}	-0.001 (-0.006)	0.849	0.001 (-0.009)	0.934	-0.004(0.012)	0.730
rs9976876(21:36970350)	T/G	-0.038(0.006)	8.01×10^{-11}	0.01 (-0.006)	0.086	0.011 (-0.009)	0.255	0.003(0.012)	0.833
rs447911(3:41127046)	C/G	0.071(0.006)	6.29×10^{-36}	0.005 (-0.006)	0.362	0.000 (-0.009)	0.968	0.026(0.012)	0.025
rs11934731(4:88831249)	A/G	-0.067(0.006)	8.39×10^{-29}	-0.011 (-0.006)	0.052	-0.007 (-0.01)	0.500	-0.011(0.013)	0.396
rs818427(5:112221869)	T/C	0.034(0.006)	2.37×10^{-08}	0.016 (-0.006)	0.008	0.032 (-0.01)	0.001	0.008(0.012)	0.530
rs11745493(5:122847622)	A/G	0.045(0.007)	7.75×10^{-12}	0.002 (-0.006)	0.723	0.013 (-0.011)	0.215	0.020(0.013)	0.138
rs7728694(5:88288341)	T/G	-0.050(0.006)	1.30×10^{-17}	-0.017 (-0.006)	0.002	-0.034 (-0.009)	0.000	0.002(0.012)	0.893
rs13204965(6:127167072)	A/C	0.062(0.007)	1.02×10^{-18}	-0.012 (-0.006)	0.058	-0.034 (-0.011)	0.001	0.020(0.013)	0.136
rs6557155(6:151910126)	T/G	-0.075(0.006)	2.56×10^{-37}	0.007 (-0.006)	0.222	-0.001 (-0.01)	0.911	0.002(0.012)	0.866
rs7741085(6:44636919)	T/C	0.042(0.006)	1.51×10^{-13}	-0.029 (-0.006)	0.000	-0.023 (-0.009)	0.013	-0.070(0.012)	0.000
rs12534510(7:120730944)	A/C	-0.040(0.006)	3.15×10^{-12}	-0.002 (-0.006)	0.714	-0.001 (-0.009)	0.920	-0.025(0.012)	0.030
rs3801387(7:120974765)	A/G	-0.135(0.006)	1.15×10^{-100}	-0.007 (-0.006)	0.242	-0.005 (-0.01)	0.661	-0.016(0.013)	0.208
rs757138(7:27989403)	T/G	-0.035(0.006)	3.33×10^{-08}	-0.004 (-0.006)	0.545	-0.022 (-0.011)	0.033	0.009(0.013)	0.513
rs73305797(7:30997087)	A/T	0.042(0.007)	2.40×10^{-10}	-0.001 (-0.006)	0.915	-0.024 (-0.011)	0.023	0.008(0.013)	0.531
rs34102936(7:38142840)	A/G	0.047(0.006)	1.87×10^{-16}	0.006 (-0.006)	0.309	0.014 (-0.009)	0.147	0.019(0.012)	0.114
rs1548607(7:50901491)	A/G	0.036(0.007)	4.18×10^{-08}	0.006 (-0.006)	0.350	0.009 (-0.01)	0.371	0.023(0.012)	0.060
rs6465511(7:96134115)	C/G	-0.074(0.006)	1.03×10^{-34}	-0.014 (-0.006)	0.017	0.000 (-0.01)	0.969	-0.040(0.012)	0.001
rs6960249(7:96660132)	T/G	0.033(0.006)	1.45×10^{-08}	0.012 (-0.006)	0.026	0.016 (-0.009)	0.089	0.015(0.012)	0.211
rs11995824(8:120012700)	C/G	0.068(0.006)	1.06×10^{-31}	0.006 (-0.006)	0.317	0.015 (-0.009)	0.106	-0.019(0.012)	0.113
rs10901216(9:133471891)	A/G	-0.047(0.006)	5.53×10^{-15}	0.006 (-0.006)	0.299	0.010 (-0.010)	0.311	0.013(0.012)	0.273

BMD bone mineral density; *OA* osteoarthritis; *FN* femoral neck; *LS* lumbar spine; *TB* total body.

Table S2. Mendelian randomization estimates for bone mineral density on osteoarthritis in alternative summary data.

Exposure	Outcome	No. of IVs	MR results			Heterogeneity tests
			Method	*Beta* (95% CI)	*P*-value	Cochran's Q (*P*)
FN-BMD	OA	36	Simple median	0.058 (0.001, 0.117)	**0.047**	19.736 (0.712)
			Weighted median	0.060 (0.005, 0.117)	**0.033**	
			IVW	0.059 (0.019, 0.100)	**0.003**	
			MR-Egger	0.078 (-0.036, 0.204)	0.188	
			MR-Egger intercept	-0.001 (-0.007, 0.005)	0.743	
LS-BMD	OA	33	Simple median	0.088 (0.030, 0.147)	**0.002**	14.517 (0.803)
			Weighted median	0.081 (0.027, 0.137)	**0.003**	
			IVW	0.075 (0.035, 0.115)	**0.001**	
			MR-Egger	0.058 (-0.063, 0.192)	0.363	
			MR-Egger intercept	0.001 (-0.006, 0.008)	0.787	
TB-BMD	OA	45	Simple median	0.035 (-0.016, 0.089)	0.185	27.289 (0.608)
			Weighted median	0.028 (-0.021, 0.081)	0.362	
			IVW	0.024 (-0.009, 0.058)	0.154	
			MR-Egger	-0.003 (-0.088, 0.090)	0.949	
			MR-Egger intercept	0.001 (-0.003, 0.006)	0.572	

BMD bone mineral density; *OA* osteoarthritis; *FN* femoral neck; *LS* lumbar spine; *TB* total body; *IVs* instrumental variables; *IVW* inverse variance weighted.

Table S3. Results of two-sample Mendelian randomization analyses of osteoarthritis on bone mineral density.

SNP (position)	Effect allele/ Other allele	SNP & OA		SNP & BMD associations					
				Total body BMD		Femoral neck BMD		Lumber spine BMD	
		Beta (SE)	*P*	*Beta* (SE)	*P*	*Beta* (SE)	*P*	*Beta* (SE)	*P*
**OA at any sites**									
rs2785988 (1:219744138)	A/C	0.039 (0.007)	1.974×10^{-09}	-0.006 (0.006)	0.367	0.002 (0.008)	0.840	-0.007 (0.010)	0.513
rs10218792 (1:245750932)	G/T	0.039 (0.007)	2.025×10^{-08}	0.000 (0.007)	0.994	0.003 (0.008)	0.722	0.017 (0.010)	0.083
rs11031191 (11:30774280)	T/G	0.030 (0.007)	1.415×10^{-08}	0.004 (0.006)	0.564	0.002 (0.008)	0.789	0.007 (0.009)	0.481
rs1149620 (11:76506572)	T/A	0.039 (0.007)	6.926×10^{-10}	-0.008 (0.006)	0.156	0.001 (0.008)	0.860	-0.001 (0.009)	0.896
rs317630 (12:69637847)	T/C	0.039 (0.007)	1.973×10^{-08}	-0.004 (0.006)	0.505	-0.006 (0.008)	0.502	0.000 (0.010)	0.961
rs2171126 (12:94167220)	T/C	0.030 (0.007)	9.066×10^{-10}	0.014 (0.006)	0.014	0.011 (0.008)	0.144	-0.011 (0.009)	0.209
rs35206230 (15:75097780)	T/C	0.039 (0.005)	1.478×10^{-12}	0.006 (0.006)	0.298	-0.009 (0.008)	0.283	0.002 (0.009)	0.875
rs1126464 (16:89704365)	G/C	0.039 (0.007)	1.555×10^{-10}	-0.006 (0.008)	0.463	0.001 (0.009)	0.955	-0.012 (0.010)	0.260
rs10502437 (18:20970706)	G/A	0.030 (0.005)	2.501×10^{-08}	-0.001 (0.006)	0.825	0.018 (0.008)	0.022	-0.012 (0.009)	0.192
rs1560707 (19:10750738)	T/G	0.039 (0.005)	1.348×10^{-13}	-0.005 (0.006)	0.443	-0.013 (0.008)	0.105	-0.013 (0.009)	0.171
rs75621460 (19:41833784)	A/G	0.148 (0.018)	1.623×10^{-15}	0.027 (0.025)	0.268	0.032 (0.026)	0.232	0.066 (0.031)	0.039
rs62182810 (2:204387482)	A/G	0.030 (0.007)	1.65×10^{-09}	-0.020 (0.006)	0.001	-0.004 (0.008)	0.579	0.004 (0.009)	0.671
rs2061027 (2:33434336)	A/G	0.039 (0.005)	3.157×10^{-13}	-0.004 (0.006)	0.499	0.007 (0.008)	0.362	0.006 (0.009)	0.484
rs3771501 (2:70717653)	A/G	0.049 (0.007)	4.236×10^{-16}	0.006 (0.006)	0.316	-0.002 (0.008)	0.818	-0.011 (0.009)	0.234
rs2248393 (20:33926103)	C/G	0.039 (0.007)	3.731×10^{-14}	0.000 (0.006)	0.961	0.019 (0.009)	0.055	0.021 (0.011)	0.055

rs62262139 (3:50022049)	A/G	0.039 (0.005)	9.093×10^{-11}	0.013 (0.012)	0.258	-0.018 (0.009)	0.052	-0.007 (0.010)	0.481
rs13107325 (4:103188709)	T/C	0.095 (0.012)	8.293×10^{-19}	0.014 (0.012)	0.255	-0.014 (0.014)	0.329	-0.027 (0.016)	0.093
rs1913707 (4:13039440)	A/G	0.039 (0.007)	2.525×10^{-10}	-0.003 (0.006)	0.563	-0.005 (0.008)	0.525	-0.006 (0.009)	0.505
rs798726 (4:1685211)	C/T	0.039 (0.007)	2.174×10^{-09}	-0.003 (0.007)	0.637	-0.004 (0.009)	0.696	-0.022 (0.011)	0.056
rs34811474 (4:25408838)	G/A	0.039 (0.005)	8.588×10^{-09}	0.011 (0.008)	0.177	0.002 (0.009)	0.797	0.015 (0.011)	0.186
rs115740542 (6:26123502)	C/T	0.058 (0.010)	1.253×10^{-09}	0.003 (0.012)	0.801	0.000 (0.016)	0.986	-0.004 (0.019)	0.846
rs2856821 (6:33046742)	T/C	0.039 (0.007)	2.705×10^{-08}	0.015 (0.008)	0.063	-0.007 (0.010)	0.455	-0.010 (0.012)	0.404
rs12154055 (6:44449697)	G/A	0.030 (0.005)	1.934×10^{-11}	0.007 (0.006)	0.231	0.006 (0.008)	0.432	-0.017 (0.009)	0.072
rs330050 (8:9087679)	G/C	0.039 (0.005)	8.548×10^{-15}	-0.021 (0.006)	0.000	-0.025 (0.008)	0.002	-0.031 (0.009)	0.001
rs919642 (9:116911147)	T/A	0.049 (0.005)	1.339×10^{-08}	-0.011 (0.007)	0.118	0.024 (0.009)	0.007	0.022 (0.010)	0.042
rs10974438 (9:4291928)	A/C	0.030 (0.007)	2.366×10^{-08}	-0.004 (0.006)	0.481	0.008 (0.008)	0.346	0.006 (0.009)	0.540
rs528981060 (22:43662241)	A/G	0.519 (0.094)	2.366×10^{-08}	----------------	-------	--------------	------	0.191 (0.102)	0.068
**Knee OA**									
rs56116847 (12:123835233)	A/G	0.058 (0.010)	3.194×10^{-10}	0.011 (0.007)	0.111	0.001 (0.009)	0.895	0.008 (0.010)	0.460
rs4775006 (15:58215727)	A/C	0.058 (0.010)	8.400×10^{-10}	-0.003 (0.006)	0.619	-0.002 (0.008)	0.781	-0.025 (0.009)	0.006
rs6499244 (16:69735271)	A/T	0.058 (0.010)	3.877×10^{-11}	-0.022 (0.006)	0.000	0.005 (0.008)	0.556	-0.007 (0.009)	0.462
rs8067763 (17:70012939)	G/A	0.058 (0.010)	2.386×10^{-09}	-0.014 (0.006)	0.015	0.033 (0.008)	0.000	0.001 (0.010)	0.920
rs12470967 (2:192671981)	A/G	0.058 (0.010)	1.498×10^{-08}	0.012 (0.006)	0.054	0.009 (0.008)	0.267	0.007 (0.009)	0.424
rs17567417 (2:33430603)	G/C	0.068 (0.010)	1.959×10^{-12}	0.003 (0.006)	0.623	0.006 (0.008)	0.406	0.008 (0.009)	0.389
rs143384 (20:34025756)	A/G	0.095 (0.009)	4.773×10^{-23}	0.002 (0.006)	0.802	0.010 (0.008)	0.200	0.024 (0.009)	0.008
rs35611929 (5:77467824)	A/G	0.058 (0.010)	1.207×10^{-08}	0.003 (0.006)	0.665	-0.012 (0.008)	0.135	-0.001 (0.009)	0.885
rs9277552 (6:33055501)	C/T	0.068 (0.012)	1.969×10^{-08}	-0.016 (0.008)	0.060	-0.005 (0.014)	0.725	-0.024 (0.016)	0.159

rs1078301 (9:116909146)	T/A	0.068 (0.010)	1.269×10^{-10}	-0.010 (0.007)	0.116	0.021 (0.009)	0.017	0.019 (0.010)	0.071
**Hip OA**									
rs4338381 (1:103572927)	A/G	0.095 (0.014)	4.371×10^{-15}	0.009 (0.006)	0.147	-0.009 (0.008)	0.246	-0.010 (0.009)	0.279
rs12040949 (1:150447462)	C/T	0.068 (0.012)	2.835×10^{-08}	-0.008 (0.006)	0.201	-0.007 (0.008)	0.357	-0.012 (0.009)	0.214
rs11583641 (1:183906245)	C/T	0.077 (0.012)	5.575×10^{-10}	-0.003 (0.006)	0.610	-0.004 (0.008)	0.664	-0.001 (0.010)	0.931
rs2785988 (1:219744138)	A/C	0.086 (0.012)	7.304×10^{-11}	-0.006 (0.006)	0.367	0.002 (0.008)	0.840	-0.007 (0.010)	0.513
rs10896015 (11:65323725)	G/A	0.077 (0.014)	2.735×10^{-09}	0.015 (0.007)	0.024	0.029 (0.009)	0.001	0.020 (0.010)	0.044
rs34419890 (11:66501624)	T/C	0.122 (0.020)	1.985×10^{-08}	0.018 (0.013)	0.152	0.042 (0.016)	0.009	0.008 (0.018)	0.655
rs11059094(12:122606837)	T/C	0.077 (0.012)	7.378×10^{-11}	0.013 (0.006)	0.029	0.021 (0.008)	0.007	0.018 (0.009)	0.049
rs10492367 (12:28014970)	T/G	0.148 (0.015)	1.248×10^{-24}	0.004 (0.007)	0.628	-0.046 (0.010)	0.000	-0.008 (0.011)	0.479
rs79056043 (12:59289598)	G/A	0.166 (0.026)	1.33×10^{-09}	-0.034 (0.013)	0.009	0.015 (0.017)	0.402	0.017 (0.020)	0.396
rs12901372 (15:67370506)	C/G	0.077 (0.012)	3.459×10^{-11}	0.020 (0.006)	0.001	-0.003 (0.008)	0.733	-0.026 (0.009)	0.004
rs62063281 (17:44038785)	G/A	0.095 (0.014)	5.298×10^{-12}	0.019 (0.009)	0.025	-0.026 (0.011)	0.021	-0.052 (0.014)	0.000
rs7222178 (17:59652282)	A/T	0.095 (0.014)	3.775×10^{-11}	0.012 (0.008)	0.110	0.014 (0.009)	0.140	0.005 (0.011)	0.651
rs4252548 (19:55879672)	T/C	0.278 (0.041)	1.956×10^{-12}	-0.040 (0.027)	0.141	0.077 (0.035)	0.031	0.029 (0.039)	0.467
rs7571789 (2:70714793)	T/C	0.086 (0.012)	3.26×10^{-14}	0.006 (0.006)	0.283	-0.001 (0.008)	0.934	-0.011 (0.009)	0.231
rs2836618 (21:40048295)	A/G	0.086 (0.014)	3.202×10^{-11}	-0.011 (0.006)	0.096	-0.011 (0.009)	0.208	-0.003 (0.010)	0.749
rs3774355 (3:52817778)	A/G	0.086 (0.012)	8.201×10^{-14}	0.007 (0.006)	0.238	-0.014 (0.008)	0.092	0.012 (0.009)	0.212
rs1913707 (4:13039440)	A/G	0.077 (0.012)	2.962×10^{-11}	-0.003 (0.006)	0.563	-0.005 (0.008)	0.525	-0.006 (0.009)	0.505
rs798748 (4:1716770)	C/T	0.068 (0.012)	2.50×10^{-09}	0.001 (0.006)	0.900	0.012 (0.008)	0.115	0.000 (0.009)	0.964
rs115740542 (6:26123502)	C/T	0.122 (0.022)	1.604×10^{-08}	0.003 (0.012)	0.801	0.000 (0.016)	0.986	-0.004 (0.019)	0.846
rs2396502 (6:45357699)	C/A	0.086 (0.012)	2.118×10^{-12}	0.027 (0.006)	0.000	0.010 (0.008)	0.221	0.029 (0.009)	0.002

rs80287694 (6:55636940)	G/A	0.113 (0.018)	2.66×10^{-09}	0.012 (0.009)	0.171	0.001 (0.012)	0.929	-0.006 (0.013)	0.679
rs12209223 (6:76164589)	A/C	0.157 (0.017)	3.883×10^{-16}	0.000 (0.010)	0.992	-0.016 (0.013)	0.213	0.005 (0.015)	0.752
rs2929451 (8:9085295)	A/T	0.068 (0.012)	3.107×10^{-09}	0.022 (0.006)	0.000	0.027 (0.008)	0.001	0.029 (0.009)	0.003
rs1330349 (9:117840742)	C/G	0.077 (0.012)	4.099×10^{-11}	0.004 (0.006)	0.488	-0.006 (0.008)	0.427	-0.013 (0.009)	0.158
rs34687269 (9:119484132)	A/T	0.086 (0.012)	1.671×10^{-12}	0.006 (0.006)	0.290	0.001 (0.008)	0.898	-0.013 (0.009)	0.164
rs62578127 (9:129386860)	C/T	0.086 (0.012)	2.771×10^{-12}	-0.004 (0.006)	0.525	-0.005 (0.008)	0.515	-0.001 (0.009)	0.900

BMD bone mineral density; *OA* osteoarthritis; *FN* femoral neck; *LS* lumbar spine; *TB* total body.

TableS4. Subgroup analysis using Mendelian randomization estimates for bone mineral density on osteoarthritis by age.

Exposure	Outcome	No. of IVs	Method	MR results (30-45 years)		Heterogeneity tests	MR results (45-60 years)		Heterogeneity tests	MR results (Above 60 years)		Heterogeneity tests
				Beta (SE)	*P*-value	Cochran's Q (*P*)	*Beta* (SE)	*P*-value	Cochran's Q (*P*)	*Beta* (SE)	*P*-value	Cochran's Q (*P*)
TB-BMD	OA at any sites	43	Weighted median	0.067(0.035)	0.061	12.450 (0.087)	0.112(0.034)	**0.002**	32.293 (**0.020**)	0.125(0.036)	**0.001**	64.423 (**0.001**)
			IVW	0.082(0.033)	**0.017**		0.154(0.030)	**0.001**		0.113(0.043)	**0.013**	
			MR-Egger	-0.051(0.161)	0.757		0.003(0.105)	0.978		0.240(0.168)	0.216	
			MR-Egger intercept	0.014 (0.017)	0.435		0.013 (0.009)	0.181		-0.010 (0.014)	0.515	
	KOA		Weighted median	0.021(0.041)	0.606	19.684 (**0.006**)	0.100(0.043)	**0.027**	45.047 (**0.001**)	0.041(0.043	0.348	45.377 (**0.001**)
			IVW	0.073(0.049)	0.152		0.178(0.043)	**0.001**		0.108(0.044)	**0.020**	
			MR-Egger	-0.022(0.248)	0.933		-0.018(0.151)	0.905		0.256(0.172)	0.202	
			MR-Egger intercept	0.010 (0.026)	0.717		0.017 (0.013)	0.224		-0.011 (0.015)	0.462	
	HOA		Weighted median	0.115(0.056)	0.053	8.103 (0.230)	0.133(0.052)	**0.015**	36.447 (**0.006**)	0.163(0.060)	**0.011**	63.515 (**0.001**)
			IVW	0.116(0.048)	**0.021**		0.132(0.047)	**0.009**		0.134(0.065)	0.054	
			MR-Egger	-0.067(0.222)	0.767		0.054(0.171)	0.758		0.221(0.256)	0.444	
			MR-Egger intercept	0.019 (0.023)	0.439		0.007 (0.015)	0.673		-0.006 (0.022)	0.766	

BMD bone mineral density; *OA* osteoarthritis; *FN* femoral neck; *LS* lumbar spine; *TB* total body; *IVs* instrumental variables; *IVW* inverse variance weighted.
